# RiemannInfer: improving transformer inference through Riemannian geometry

**DOI:** 10.1038/s41598-026-37328-x

**Published:** 2026-01-29

**Authors:** Runze Mao, Zhengyuan Zhang, Mengyao Yang, Hui Xie, Shengjun Wei, Changzhen Hu

**Affiliations:** 1 School of Cyberspace Science and Technology, Beijing Institute of Technology, No. 5, South Street, 100081 Beijing, Asia China; 2 School of Computer Science and Technology, Beijing Institute of Technology, No. 5, South Street, 100081 Beijing, Asia China; 3 Beijing Key Laboratory of Software Security Engineering Technology, 100081 Beijing, Asia China

**Keywords:** Riemannian Geometry, Large Language Models, Topological Dimensionality Reduction, Reasoning Path Planning, Inference Work Calculation, Mathematics and computing, Physics

## Abstract

Understanding the reasoning process of Large Language Models(LLMs) is crucial for interpreting their decision-making mechanisms. However, existing methods primarily rely on conditional probability distributions within the model, resulting in computationally inefficient reasoning processes and difficulties in constructing rigorous logical chains. This paper presents RiemannInfer, an innovative computational framework based on Riemannian geometry for optimizing LLM reasoning paths. Our approach stems from a key observation: the hidden states formed through attention mechanisms and contextual encoding in the Transformer can be represented as high-dimensional vector spaces that encapsulate global dependencies captured by the model. Building on this insight, we first apply topology-preserving dimensionality reduction to these hidden states, then construct a Riemannian manifold structure utilizing attention distribution features. Notably, We discover a close relationship between the attention mechanism to the geodesics and the curvature of Riemannian manifolds. Based on this foundation, we implement efficient inference work calculation through geodesic and curvature to realize reasoning path planning, which not only significantly enhances inference efficiency but also provides geometric interpretations of model decision processes. Extensive experiments on various models, including LLaMA, GPT-4, and DeepSeek, demonstrate that RiemannInfer significantly improves the reasoning accuracy of LLMs and exhibits excellent effectiveness and robustness. This research provides a novel geometric perspective for enhancing the interpretability and efficient reasoning of LLMs.

## Introduction

Large Language Models (LLMs) have revolutionized natural language processing, demonstrating remarkable capabilities across diverse tasks ranging from text generation to complex reasoning^1^. Models such as GPT-4, LLaMA, and PaLM have achieved unprecedented performance levels, approaching human-like reasoning in many domains^2,3^. However, despite their impressive capabilities, understanding the internal reasoning processes of these models remains a significant challenge. This challenge not merely enhances the interpretability, but it has profound implications for model reliability, trustworthiness, and deployment in critical applications^4,5^.

The dominant paradigm for interpreting LLMs’ reasoning has focused primarily on analyzing conditional probability distributions within the models^6–8^. While this approach has provided valuable insights, it suffers from two fundamental limitations. First, reasoning processes based on these methods tend to be computationally inefficient, often requiring extensive sampling and prompting techniques that significantly increase latency and resource consumption^9^. Second, these methods struggle to construct rigorous logical chains that can reliably explain the model’s decision-making process, particularly in complex reasoning tasks^10,11^.

These limitations have motivated researchers to explore alternative perspectives for understanding and optimizing LLM reasoning. Geometric approaches, which have proven powerful in other areas of machine learning^12,13^, offer a promising direction. By conceptualizing the internal representations of LLMs as structured geometric spaces, we can potentially develop more efficient and interpretable methods for reasoning path optimization. This geometric perspective is particularly appealing because it provides a natural framework for understanding the continuous, high-dimensional spaces in which LLMs operate^14,15^.

In this paper, we propose RiemannInfer, a novel computational methodology that leverages principles from Riemannian geometry to optimize reasoning paths in LLMs. Our approach is founded on a key observation: the hidden states formed through attention mechanisms and contextual encoding in the Transformer can be represented as high-dimensional vector spaces that encapsulate the global dependencies captured by the model. This observation allows us to reformulate the reasoning process as a path-finding problem on a carefully constructed Riemannian manifold.

**Contributions.** Overall, key contributions are three-fold: **(1.)** Topological Dimensionality Reduction. We apply Uniform manifold approximation and projection (Umap) to reduce the dimensionality of the hidden states in the Transformer, which reduces the dimensionality and preserves the topological structure efficiently while maintaining the fundamental semantic relationships. **(2.)** Riemannian Manifold Construction. We propose a novel method for constructing Riemannian manifolds from attention distribution features, including an adaptive metric tensor learning algorithm that accurately reflects the semantic space characteristics of different models. **(3.)** Reasoning Path Planning. We propose an efficient reasoning path planning method by calculating the inference work by measuring geodesics and curvature on Riemannian manifolds, providing both geometric interpretations of model decisions and significant improvements in inference efficiency.

We conduct extensive experiments across multiple LLM architectures (LLaMA, GPT-4, and Deepseek) and reasoning tasks, demonstrating that RiemannInfer significantly improves the reasoning accuracy of LLMs and exhibits excellent effectiveness and robustness.

Our work contributes to the growing body of research on LLM interpretability and efficiency, offering a novel geometric perspective that bridges theoretical insights from differential geometry with practical improvements in model performance. By reformulating reasoning as navigation through a geometric space, we not only enhance computational accuracy but also provide new tools for visualizing and understanding the decision processes of these increasingly important models.

The remainder of this paper is organized as follows: Section Related works reviews related works in LLM reasoning methods and applications of geometric approaches in machine learning. Section Theoretical background provides the necessary theoretical background on Riemannian geometry and Transformer representations. Section Implementation of RiemannInfer details our RiemannInfer methodology, including dimensionality reduction, manifold construction, and reasoning path planning. Section Experiments presents our experimental setup and results, respectively. Finally, Section Conclusion concludes the paper and looks forward to future work.

## Related works

Understanding the reasoning processes of LLMs holds extraordinary significance for interpreting model decisions. The capabilities of reasoning models developed by researchers have progressively advanced through architectural innovations and paradigm shifts in training methodologies. The Transformer architecture proposed by Vaswani et al.^16^ established foundational principles for sequence modeling, and subsequent improvements such as sparse attention mechanisms proposed by Beltagy et al.^17^ significantly enhanced efficiency in long-context reasoning tasks. Explicit modeling of reasoning steps, exemplified by Chain-of-Thought (CoT)^6^ prompting and Program-Aided Language models(PAL)^18^, has enabled human-like logical inference in mathematical and symbolic domains. Building upon CoT, Tree-of-Thought (ToT)^7^ and Graph-of-Thought (GoT)^8^ frameworks have recently emerged to tackle complex multi-step reasoning. Furthermore, hybrid architectures like Retrieval-Augmented models^19^ and Neuro-Symbolic frameworks^20^ have been developed to integrate external knowledge bases, thereby improving factual consistency in model outputs.

Manifold geometry has been demonstrated as a powerful tool for encoding hierarchical and non-Euclidean geometric structures. Hyperbolic neural networks^21^ and Poincaré embeddings^22^ have exhibited exceptional performance in hierarchical representation learning, later extended to graph neural networks by Chami et al.^23^. Pan et al.^24^ formalized attention mechanisms on Riemannian manifolds, enabling the capture of latent geometric relationships in high-dimensional embeddings of the Transformer. To enhance training stability and generalization capability, optimization frameworks such as Umap^25^ and manifold adaptation methods^26^ have been progressively developed.

The synergy between manifold geometry and LLMs now stands at the forefront of machine learning research. Wang et al.^27^ proposed manifold-based contrastive learning to regularize the latent space of pre-trained models, effectively mitigating hallucination phenomena in generation tasks. Concurrently, Li et al.^28^ introduced curvature-regularized fine-tuning, which aligns the geometric properties of model representations with task-specific manifolds, achieving state-of-the-art results in multi-hop reasoning benchmarks. These advancements substantiate that manifold geometry can provide theoretical underpinnings for enhancing LLM reasoning capabilities.

## Theoretical background

This section establishes the theoretical foundations necessary for understanding the RiemannInfer framework. We first introduce the core concepts of Riemannian geometry, including Riemannian manifolds, metric tensors, geodesics, and curvatures. We then analyze the mathematical properties of representation spaces in the Transformer and finally reinterpret attention mechanisms from a geometric perspective. These theoretical foundations provide the mathematical framework for our RiemannianInfer methodology presented in Section Implementation of RiemannInfer.

### Foundations of Riemannian geometry

Riemannian geometry, as a significant branch of differential geometry, provides powerful mathematical tools for studying non-Euclidean spaces. This subsection briefly introduces the core concepts of Riemannian geometry that will serve as the theoretical basis for analyzing the computational structure of the Transformer.

#### Definition 1

(*Riemannian Manifold*) A Riemannian manifold $\mathcal {M}$ is a smooth manifold where each tangent space $T_p\mathcal {M}$ at point $p \in \mathcal {M}$ is equipped with an inner product $g_p$ that varies smoothly concerning *p*.

The essential characteristic of Riemannian manifolds lies in their locally Euclidean nature while potentially exhibiting complex curvature globally. At a point *p* on the manifold, the tangent space $T_p\mathcal {M}$ consists of all tangent vectors to curves passing through *p*. If the manifold has dimension *n*, then the tangent space is also *n*-dimensional. In a local coordinate system $(x^1, x^2,..., x^n)$, the basis for the tangent space can be represented as ${\frac{\partial }{\partial x^1}|_p, \frac{\partial }{\partial x^2}|_p,..., \frac{\partial }{\partial x^n}|_p}$.

#### Definition 2

(*Riemannian Metric Tensor*) A Riemannian metric is a smooth, symmetric, positive-definite covariant tensor field *g* that defines an inner product on the tangent space $T_p\mathcal {M}$ at each point $p \in \mathcal {M}$.

In local coordinates $x_i$, the metric tensor can be represented as a matrix $[g_{ij}]$ where:

1 $$\begin{aligned} g = g_{ij}(x) \, dx^i \otimes dx^j \end{aligned}$$

The components $g_{ij}(x)$ satisfy the following properties:

1. Symmetry: $$g_{ij}=g_{ji}$$.
2. Positive Definiteness: For any non-zero tangent vector $v \in T_p\mathcal {M}$, the inner product satisfies: $g_p(v,v)>0$.

#### Definition 3

(*Geodesic*) A geodesic is a curve on a Riemannian manifold that locally minimizes length and satisfies the geodesic equation:

2 $$\begin{aligned} \frac{d^2 x^i}{d t^2}+\Gamma _{j k}^i \frac{d x^j}{d t} \frac{d x^k}{d t}=0 \end{aligned}$$

Where $\Gamma ^i_{jk}$ are the Christoffel symbols determined by the metric tensor:

3 $$\begin{aligned} \Gamma _{j k}^i=\frac{1}{2} g^{i l}\left( \frac{\partial g_{l j}}{\partial x^k}+\frac{\partial g_{l k}}{\partial x^j}-\frac{\partial g_{j k}}{\partial x^I}\right) \end{aligned}$$

Geodesic plays a role analogous to straight lines in Euclidean space, representing the “shortest paths” between points on the manifold. Closely related to geodesics is the concept of Parallel Transport, which makes parallel transport tangent vectors along curves.

#### Definition 4

(*Parallel Transport*) Parallel transport along a curve $\gamma$ is a linear map $P_{\gamma , t_0, t_1}: T_{\gamma (t_0)}\mathcal {M} \rightarrow T_{\gamma (t_1)}\mathcal {M}$ that preserves vector lengths and angles, satisfying the parallel transport equation:

4 $$\begin{aligned} \frac{D V^i}{d t}=\frac{d V^i}{d t}+\Gamma _{j k}^i V^j \frac{d x^k}{d t}=0 \end{aligned}$$

#### Definition 5

(*Curvature*) The curvature of a Riemannian manifold describes the degree to which it deviates from Euclidean space, represented as a fourth-order tensor *R*. In local coordinates, *r* is expressed as

5 $$\begin{aligned} R_{j k l}^i=\frac{\partial \Gamma _{j l}^j}{\partial x^k}-\frac{\partial \Gamma _{j k}^j}{\partial x^l}+\Gamma _{m k}^i \Gamma _{j l}^m-\Gamma _{m l}^i \Gamma _{j k}^m \end{aligned}$$

These fundamental concepts of Riemannian geometry provide the framework for analyzing the geometric properties of representation spaces in the Transformer and designing geometry-based reasoning planning algorithms.

### Representation spaces in the transformer

We establish the mathematical framework that allows us to view the high-dimensional representation spaces of Transformers through the lens of differential geometry, providing theoretical foundations for transforming the hidden states of the Transformer from state spaces into Riemannian manifolds.

#### Theorem 1

(Manifold Hypothesis) *The hidden representations in the Transformer lie on a lower-dimensional Riemannian manifold* $\mathcal {M}$ *embedded in the ambient space* $\mathbb {R}^d$ *approximately, where* *d* *is the embedding dimension*.

The research by Pope et al. has confirmed that the intrinsic dimensionality of the representation space is significantly lower than that of its surrounding dimensions^29^, providing strong evidence for this hypothesis. For a Transformer model with *L* layers, we can define a sequence of manifolds ${\mathcal {M}l}{l=1}^L$, where $\mathcal {M}_l$ represents the manifold of hidden states at layer *l*. Each manifold $\mathcal {M}_l$ can be characterized by:

**(1.)**: An embedding map $\phi _l: \mathcal {M}_l \rightarrow \mathbb {R}^d$ that associates each abstract point on the manifold with its coordinate representation.
**(2.)**: A Riemannian metric $g_l$ that defines the local geometry at each point $p \in \mathcal {M}_l$.

To operationalize the manifold perspective, we need methods to approximate the tangent spaces at various points on the manifold.

#### Definition 6

(*Tangent Space Approximation*) At a hidden state $h \in \mathcal {M}_l$, the tangent space $T_h\mathcal {M}_l$ can be approximated using local principal component analysis (PCA) on a neighborhood of points around *h*.

Formally, given a neighborhood $\mathcal {N}(h) = {h_1, h_2,..., h_k}$ of *h*, we compute the centered data matrix $X = [h_1 - \bar{h}, h_2 - \bar{h},..., h_k - \bar{h}]$ where $\bar{h} = \frac{1}{k}\sum _{i=1}^k h_i$. The singular value decomposition $X = U\Sigma V^T$ provides the basis vectors *U* for the tangent space approximation.

The transformation between consecutive layers in a Transformer can be interpreted geometrically.

#### Theorem 2

(Layer Mapping) *The transformation from layer* *l* *to layer* $l+1$ *in a Transformer defines a smooth map* $f_l: \mathcal {M}l \rightarrow \mathcal {M}{l+1}$ *between manifolds*.

This mapping can be decomposed into:

**(1.)**: Self-attention operation: $f_l^{attn}: \mathcal {M}_l \rightarrow \mathcal {M}_l^{int}$
**(2.)**: Feed-forward network: $f_l^{ffn}: \mathcal {M}l^{int} \rightarrow \mathcal {M}{l+1}$

Where $\mathcal {M}_l^{int}$ is an intermediate manifold. The differential of this mapping, $df_l: T_h\mathcal {M}l \rightarrow T{f_l(h)}\mathcal {M}_{l+1}$, describes how tangent vectors (directions) at layer *l* are transformed into tangent vectors at layer $l+1$. This differential can be approximated by:

6 $$\begin{aligned} d f_l(v) \approx \frac{f_l(h+\epsilon v)-f_l(h)}{\epsilon } \end{aligned}$$

For small $\epsilon$ and tangent vector $v \in T_h\mathcal {M}_l$.

According to the above geometric properties, the token embedding space in Transformers naturally inherits a Riemannian structure.

#### Theorem 3

(Embedding Manifold) *The token embedding space forms a Riemannian manifold* $\mathcal {E}$ *with a metric induced by the pre-training objectives that capture semantic relationships between tokens*.

The geometry of this embedding manifold is non-trivial, manifested in several properties:

1. Embeddings tend to cluster in narrow cones rather than being uniformly distributed.
2. Semantically related tokens form nested submanifolds.
3. The space exhibits non-Euclidean properties, particularly in regions of high semantic density.

The above theoretical discussion explores the spatial representation properties of the Transformer model. By embedding it into a Riemannian manifold, we can achieve more efficient reasoning computation.

### Geometric interpretation of attention mechanisms

The definition of the Riemannian metric is another key aspect in constructing the Riemannian manifold for hidden states. Attention weights implicitly represent semantic relationships between tokens, making them suitable as a foundation for building the Riemannian metric and calculating geodesic distance. The theoretical basis of this approach will be discussed and analyzed.

We first prove that the self-attention mechanism implicitly defines a Riemannian metric on the representation manifold. Consider a sequence of hidden states ${h_1, h_2,..., h_n} \subset \mathbb {R}^d$ in a Transformer, where $h_i$ represents the representation of the *i*-th token. Self-attention computes an attention weight matrix $A = [a_{ij}]$, where:

7 $$\begin{aligned} a_{i j}=\frac{\exp \left( q_i \cdot k_j / \sqrt{d_k}\right) }{\sum _{l=1}^n \exp \left( q_i \cdot k_l / \sqrt{d_k}\right) } \end{aligned}$$

Where $q_i = W_Q h_i$ and $k_j = W_K h_j$ are the query and key vectors, respectively.

#### Theorem 4

(Attention-Induced Metric) *The self-attention weight matrix* *A* *can be used to construct a valid Riemannian metric tensor* $g^A$ *on the representation manifold* $\mathcal {M}$*, with components in a local coordinate system defined as:*

8 $$\begin{aligned} g_{i j}^A(h)=\delta _{i j}-\frac{1}{2}\left( a_{i j}+a_{j i}\right) \end{aligned}$$

*Where* $\delta _{ij}$ *is the Kronecker delta function*.

To prove that $g^A$ is a valid Riemannian metric, we need to verify that it satisfies (1)symmetry and (2)positive-definiteness. Symmetry is evident since $g^A_{ij} = g^A_{ji}$. For positive-definiteness, consider any non-zero tangent vector $v \in T_h\mathcal {M}$, we have:

9 $$\begin{aligned} g^A(v, v)=\sum _{i, j} g_{i j}^A v^i v^j=\sum _i\left( v^i\right) ^2-\frac{1}{2} \sum _{i, j}\left( a_{i j}+a_{j i}\right) v^i v^j \end{aligned}$$

Since attention weights $a_{ij}$ typically satisfy $\sum _j a_{ij} = 1$ and $a_{ij} \ge 0$, it can be shown that $g^A(v,v) > 0$ for any non-zero vector *v*.

This metric tensor $g^A$ has profound geometric significance: it transforms attention weights into distance relationships in a metric space. When the attention weight between two tokens is large, their “distance” on this Riemannian manifold is small; conversely, token pairs with small attention weights are far apart on the manifold.

With the Riemannian metric $g^A$, we can define geodesic distances on the representation manifold, which represents the length of the shortest path between two points.

#### Theorem 5

(Attention Geodesic Distance) *On the Riemannian manifold induced by attention, the geodesic distance* $d_g(h_i, h_j)$ *between hidden states* $h_i$ *and* $h_j$ *is expressed as:*

10 $$\begin{aligned} d_g\left( h_i, h_j\right) =\inf _\gamma \int _0^1 \sqrt{g_{\gamma (t)}^A(\dot{\gamma }(t), \dot{\gamma }(t))} d t \end{aligned}$$

*Where* $\gamma : [0,1] \rightarrow \mathcal {M}$ *is any smooth curve connecting* $h_i$ *and* $h_j$ *with* $\gamma (0) = h_i$ *and* $\gamma (1) = h_j$.

For practical computation, we can derive an approximate relationship between geodesic distance and attention weights. For relatively close hidden states $h_i$ and $h_j$, their geodesic distance can be approximated as:

11 $$\begin{aligned} d_g\left( h_i, h_j\right) \approx \sqrt{2\left( 1-\frac{a_{i j}+a_{j i}}{2}\right) } \end{aligned}$$

Consider the linear interpolation $\gamma (t) = (1-t)h_i + th_j$ between $h_i$ and $h_j$, and compute its length under the metric $g^A$. Through first-order Taylor expansion and leveraging properties of attention weights, we arrive at the above approximation.

This result reveals a key insight: attention weights are inversely related to geodesic distances. Specifically, when $a_{ij}$ and $a_{ji}$ approach 1 (i.e., two tokens strongly attend to each other), their geodesic distance approaches 0; when attention weights approach 0, the geodesic distance approaches the maximum. This insight reveals the geometric properties of attention weights.

The problem of calculating geodesic distance involves the Euler-Lagrange equation and an efficient approximation-solving method.

#### Theorem 6

(Attention Geodesic Equations) *Under the attention-induced metric* $g^A$*, a geodesic* $\gamma (t)$ *satisfies the following second-order differential equations:*

12 $$\begin{aligned} \frac{d^2 \gamma ^k}{d t^2}+\sum _{i, j} \Gamma _{i j}^k(\gamma (t)) \frac{d \gamma ^i}{d t} \frac{d \gamma ^j}{d t}=0, \quad k=1,2, \ldots , d \end{aligned}$$

*Where* $\Gamma ^k_{ij}$ *are the Christoffel symbols associated with the metric* $g^A$*, defined as:*

13 $$\begin{aligned} \Gamma _{i j}^k=\frac{1}{2} \sum _l g^{k l}\left( \frac{\partial g_{i l}}{\partial x^j}+\frac{\partial g_{j l}}{\partial x^i}-\frac{\partial g_{i j}}{\partial x^l}\right) \end{aligned}$$

*Where* $g^{kl}$ *is the inverse of the metric tensor* $g^A$.

However, directly solving the geodesic equations is computationally expensive, especially for high-dimensional representation spaces. To address this, we propose an efficient approximation method.

#### Theorem 7

(Efficient Geodesic Approximation) *On the attention-induced Riemannian manifold, the geodesic connecting hidden states* $h_i$ *and* $h_j$ *can be efficiently approximated by the explicit formula:*

14 $$\begin{aligned} \gamma (t)=(1-t) h_i+t h_j-\frac{t(1-t)}{2} \sum _{k=1}^n\left( a_{i k}+a_{j k}\right) \left( h_k-h_i-h_j\right) \end{aligned}$$

*Where* $t \in [0,1]$*, with an approximation error of* $O(|h_i - h_j|^3)$.

Starting from the Taylor expansion of the geodesic equation, retaining terms up to the second order, and then substituting the specific form of the attention-induced metric, we can derive the above approximation formula. This approximate formula avoids the need to solve complex differential equations, opting instead for vector calculations, significantly improving the computational efficiency of calculating geodesic distances.

The multi-head attention mechanism in Transformers can be understood geometrically as defining multiple complementary metric structures on the representation manifold.

#### Theorem 8

(Multi-metric Structure of Multi-head Attention) *Let* $A^{(1)}, A^{(2)},..., A^{(H)}$ *be the attention weight matrices produced by* *H* *attention heads. Each head induces a Riemannian metric* $g^{(h)}$*. The overall multi-head attention can be viewed as a weighted combination of these metrics:*

15 $$\begin{aligned} g^{m u l t i}=\sum _{h=1}^H w_h g^{(h)} \end{aligned}$$

*Where weights* $w_h$ *are implicitly determined by the output projection matrices* $W_O^{(h)}$ *of the attention heads*.

This multi-metric structure explains the power of multi-head attention: different attention heads can capture different geometric aspects of the representation space, such as one head focusing on grammatical relationships while another focuses on semantic similarities.

In addition to the geodesics being calculable based on the attention weights, the Riemannian manifold induced by attention also possesses specific curvature properties.

#### Theorem 9

(Curvature of Attention Metric) *On the attention-induced Riemannian manifold, the scalar curvature* *R(h)* *at point* *h* *is related to the entropy of the attention distribution:*

16 $$\begin{aligned} R(h) \approx C-\alpha \sum _{j=1}^n a_{h j} \log a_{h j} \end{aligned}$$

*Where* *C* *and* $\alpha$ *are constants related to model parameters*.

This result indicates that more concentrated attention distributions (low entropy) correspond to higher curvature, while more uniform attention distributions (high entropy) correspond to lower curvature. This variation in curvature reflects the distribution of information density in the representation space and reveals the connection between information space and non-Euclidean geometric space from another perspective.

Through rigorous theoretical analysis of Riemannian manifolds, spatial representations in the Transformer, and attention mechanisms, we establish a comprehensive acceleration framework for inference based on Riemannian geometry, which provides the mathematical underpinnings for RiemannInfer.

## Implementation of RiemannInfer

**Fig. 1:**
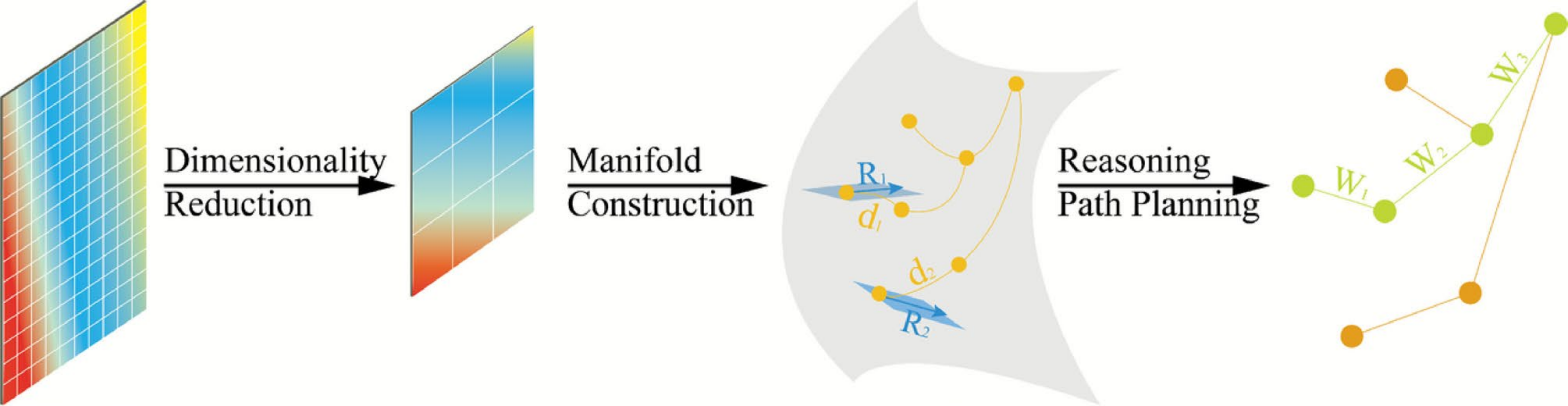
Overview of RiemannInfer.

In this section, the implementation methodology of RiemannInfer is described in detail. To enable the reasoning process of the Transformer model to be computed within a Riemannian manifold, our approach is divided into three key steps: Topological Dimensionality Reduction, Riemannian Manifold Construction, and Reasoning Path Planning, as illustrated in Fig. 1.

Through these three steps, we transform the spatial characteristics embedded in the high-dimensional hidden states of the Transformer model into a computational framework for reasoning paths. Before dimensionality reduction, we employ persistent homology analysis to capture the topological features among hidden states. Subsequently, the Umap algorithm is applied to reduce dimensionality and construct the topological structure. The core of Riemannian manifold construction lies in estimating the metric tensor using attention weights. The Riemannian manifold is further discretized to facilitate the computation of geodesics and curvature. Finally, a method for computing the optimal reasoning path is proposed based on the geodesics and curvature of state points on the Riemannian manifold.

### Topological dimensionality reduction

Before applying Umap for topological dimensionality reduction of the hidden states, the persistent homology analysis is employed to ensure that the topological structure of the hidden states is preserved as much as possible. This approach allows the reduced-dimensional representations to retain properties related to attention weights between nodes. The choice of Umap for dimensionality reduction is also motivated by its ability to maintain such relationships effectively. Each layer’s hidden states undergo the same dimensionality reduction process, thereby laying the foundation for subsequent Manifold Construction and Reasoning Path Planning tasks.

### Riemannian manifold construction

The key to defining a Riemannian manifold for topological structures lies in estimating the metric tensor, which is essential for computing geodesics and curvatures, enabling the calculation of path-planning tasks. Specifically, the metric tensor is derived from the pairwise local geometric structures among hidden states. This approach ensures the smoothness of the manifold, efficiently captures curvature, and reflects the underlying spatial complexity of the hidden states. The geodesics and curvatures of the manifold can also be quickly computed numerically using the theoretical methods from Section Theoretical background.

To ensure that the constructed manifold is both smooth and connected, we apply local smoothing techniques and enforce neighborhood connectivity constraints. Specifically, after estimating the metric tensor, we implement a k-nearest neighbor graph to guarantee that each point on the manifold maintains continuous local relationships with its neighbors. This approach mitigates discontinuities and preserves the manifold’s global structure. Local smoothing filters are further applied to the metric tensor field, which enhances the differentiability of the manifold and prevents abrupt geometric changes. Together, these steps ensure that the resulting Riemannian manifold is suitable for reliable geodesic computation and robust path planning.

### Reasoning path planning

By leveraging the construction of Riemannian manifolds, task-oriented reasoning computations are transformed from a probabilistic distribution problem in the state space into a path-planning problem on the manifold. Unlike traditional Euclidean geometry, path planning on a manifold is significantly influenced by curvature, which determines the path’s ruggedness degree between two points. Therefore, it is essential to develop accurate computational methods that account for the effects of curvature. The curvature between two points in the topological structure is calculated according to the theoretical methods presented in Section Theoretical background. Additionally, a series of techniques such as regularization and matrix pseudoinverse are employed to enhance the stability of the curvature computation.

**Fig. 2:**
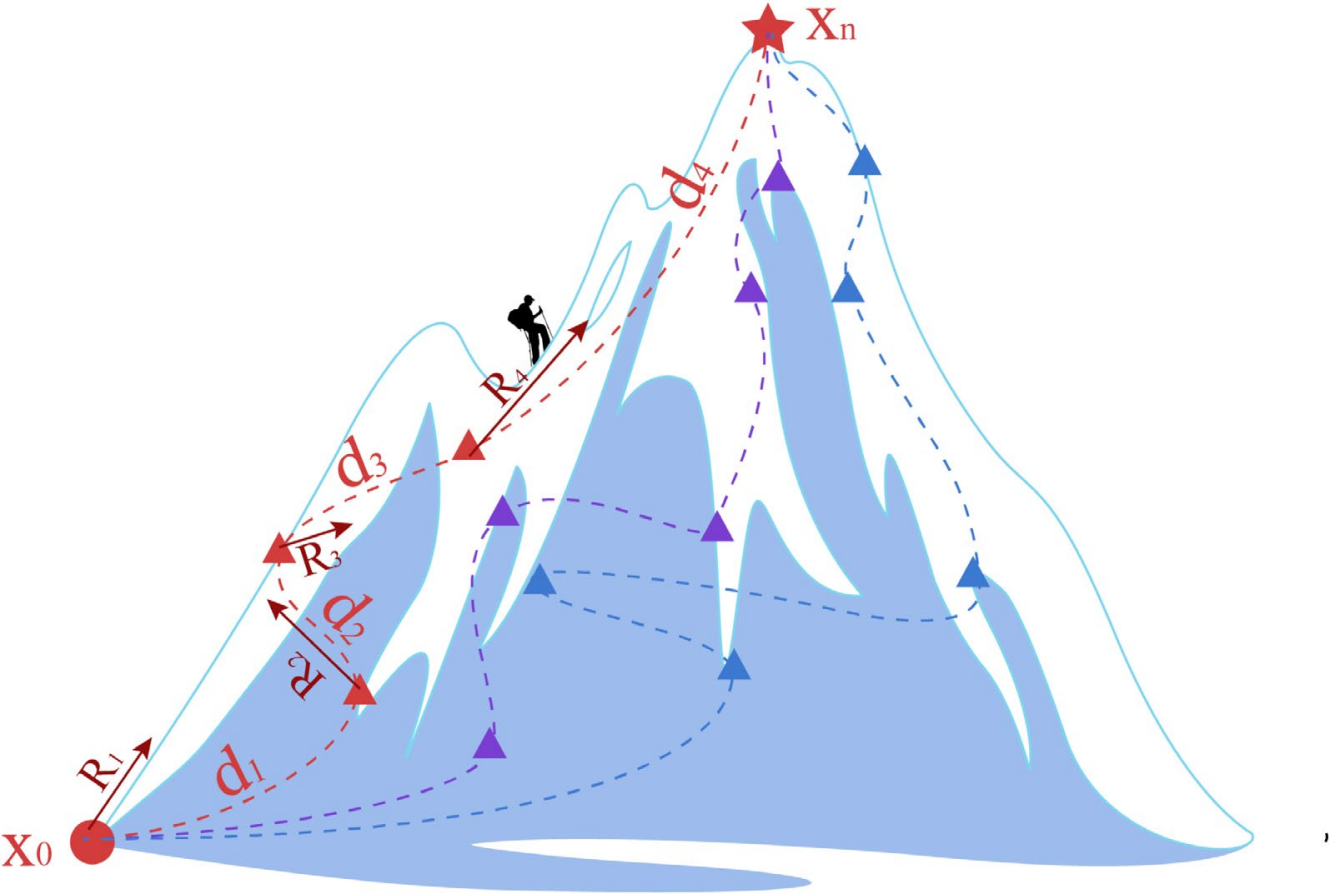
Path planning on Riemannian manifold.

We propose an innovative approach to path planning on manifolds by determining the optimal path through the computation of the work done between two points. From an information-theoretic perspective, the model’s reasoning process reflects the changes in relative information entropy between states, representing the reduction of task-specific uncertainty. Under this framework, reasoning work is identified as the driving force behind entropy changes, and its computation provides a precise metric for evaluating the value of reasoning paths. To implement this idea, we incorporate geodesics and curvature into the calculation of work done between two points on the manifold. For clarity, the detailed process of work computation is illustrated in Fig. 2.

The path-planning problem on a manifold can be conceptualized as a mountain-climbing process. A climber starts at an initial point $x_0$ and aims to reach the summit $x_n$, passing through several intermediate resting points $x_1,x_2,...,x_i,...,x_{n-1}$.The climber must strategically plan the climbing route to minimize energy expenditure while reaching the summit. In this process, both the distances between resting points and the steepness of the climbing path are critical factors. From basic physical principles, it is well understood that steeper slopes require greater force to maintain forward motion. Translating this analogy to manifolds, the rate of change in curvature effectively represents the variation in the ruggedness of the reasoning path. An increase in curvature rate implies a corresponding increase in the force required along the path, leading to greater reasoning work. Therefore, we propose the computational formula for reasoning work on Riemannian manifolds:

17 $$\begin{aligned} W=\alpha \int _{t_1}^{t_2} g_{i j}(x) \frac{\partial R(x)}{\partial x^j} \frac{d x^i}{d t} d t \end{aligned}$$

Where $\alpha$ is a constant, $g_{i j}(x)$ represents the metric tensor between two points, and $\frac{\partial R(x)}{\partial x^j}$ denotes the partial derivative of curvature along the path. In practical path-planning scenarios, based on Dijkstra’s algorithm^30^, it is sufficient to compute the minimum work between adjacent points in a discrete weighted graph at each step. Therefore, the above formula is discretized as follows:

18 $$\begin{aligned} W \approx \alpha g_{i j}\left( x_1\right) \left( \frac{R\left( x_2\right) -R\left( x_1\right) }{\Delta x_1^j}\right) \left( x_2^i-x_1^i\right) \end{aligned}$$

The computation of work along the reasoning paths is similarly refined, following an approach analogous to the curvature calculation, to ensure that the work performed on each path can be accurately determined. Meanwhile, the Dijkstra algorithm is optimized to guarantee that the optimal path is obtained in each reasoning process.

Based on the above formula and in conjunction with Dijkstra’s algorithm, the minimum work reasoning path can be efficiently planned through numerical computation. This approach fundamentally transforms the existing method of reasoning path planning based on probabilistic distributions, significantly enhancing both accuracy and efficiency. Moreover, it endows the reasoning process with a clear physical interpretation.

### Computational complexity analysis

To further substantiate the claim of high computational efficiency, we provide a theoretical analysis of the computational complexity for the main modules in RiemannInfer. Specifically:

**Topological dimensionality reduction (UMAP): **For n hidden states, UMAP performs dimensionality reduction with a time complexity of *O*(*nlogn*), which significantly reduces the data size and dimensionality for subsequent manifold operations.

**Metric tensor estimation: **The local metric tensor is estimated based on attention weights with complexity approximately *O*(*nd*), where *d* is the reduced feature dimension.

**Geodesic and curvature computation: **By utilizing explicit approximation formulas, we avoid solving high-dimensional differential equations directly. The overall complexity is $O(nd^2)$, which can be further optimized by sparsification and parallel computation.

**Reasoning path planning: **The optimal reasoning path is computed using Dijkstra’s algorithm on the discretized manifold graph, with complexity $O(E+nlogn)$, where *E* denotes the number of adjacency relations.

In summary, while operations on high-dimensional Riemannian manifolds can indeed introduce substantial computational overhead, RiemannInfer effectively mitigates this issue by employing topological dimensionality reduction and explicit approximation methods. Specifically, the use of UMAP significantly reduces the dimensionality of hidden representations, and our geodesic and curvature computations rely on efficient, locally approximated formulas rather than costly direct solutions. These strategies substantially lower the computational burden typically associated with high-dimensional manifold operations, thereby ensuring the practical scalability and efficiency of RiemannInfer for large-scale inference tasks.

## Experiments

We apply the reasoning process of RiemannInfer on LLMs and observe significant performance improvements. We also conduct out-of-domain evaluations and ablation studies, demonstrating the generalization ability and effectiveness of RiemannInfer.

### Setup

We load Llama-3-405B^31^, GPT-4o^32^, and DeepSeek-V2-400B^33^, et al. on RiemannInfer, conduct domain evaluations through GSM8k and MATH500, and use StrategyQA and AGIEval for out-of-domain evaluation. To verify the effectiveness of RiemannInfer, we constructed a geometric structure reasoning model based on the hidden states of the Transformer as a control.

#### Dataset

We select widely used reasoning benchmarks to assess both numerical and symbolic reasoning abilities comprehensively:

**GSM8K** is a grade-school-level math word problem dataset, with 7,473 training instances and 1,319 test instances, which is available at https://github.com/openai/grade-school-math.

**MATH500** is a challenging high-school math competition dataset with 7,500 training and 5,000 test questions, covering a broad range of mathematical topics and multi-step reasoning, which is available at https://huggingface.co/datasets/HuggingFaceH4/MATH-500.

**StrategyQA** is an out-of-domain dataset for evaluating generalization ability on strategy-based question answering, which is available at https://huggingface.co/mradermacher/NAT-strategy-qa-7b-GGUF.

**AGIEval** is a standard exam dataset from various fields, including college entrance exams, law school admission tests, math competitions, and lawyer qualification tests, which is available at https://huggingface.co/mradermacher/AGI-Eval-OA-Judge-GGUF.

#### Model

We conduct experiments on three representative LLMs: Llama-3-405B, GPT-4o, and DeepSeek-V2-400B. Two primary considerations guide the selection of evaluation models in our study. First, we prioritize mainstream LLMs that are widely adopted in both academic research and real-world applications. This ensures that our experimental results are relevant, reproducible, and reflective of the current state-of-the-art in natural language processing. Second, we deliberately choose models with moderate to high inference accuracy, as this enables a meaningful assessment of the impact of RiemannInfer on both performance and reasoning quality. Models with excessively high or low baseline accuracy may obscure the relative improvements brought by our method. By focusing on popular models with balanced inference capabilities, we provide a robust and generalizable evaluation framework that highlights the practical benefits and broad applicability of RiemannInfer across diverse reasoning tasks.

### Main results

The results of the in-domain evaluation are shown in Table 1, which demonstrates the effectiveness of RiemannInfer in improving the model’s mathematical reasoning performance.

**Table 1:** Model accuracy of GSM8K and MATH500.

Model	GSM8K	MATH500
Llama-3-405B	91.6%	55.2%
GPT-4o	93.2%	54.6%
DeepSeek-V2-400B	92.5%	56.8%
Ours		
**RiemannInfer, Llama-3-405B**	93.5% $\uparrow$ **1.9%**	58.4% $\uparrow$ **3.2%**
**RiemannInfer, GPT-4o**	95.8% $\uparrow$ **2.6%**	57.9% $\uparrow$ **3.3%**
**RiemannInfer, DeepSeek-V2-400B**	94.8% $\uparrow$ **2.3%**	58.9% $\uparrow$ **2.1%**

Table 1 presents the accuracy of various large language models on the GSM8K and MATH500 benchmarks. Baseline models, including Llama-3-405B, GPT-4o, and DeepSeek-V2-400B, achieved accuracy rates of 91.6%, 93.2%, and 92.5% on GSM8K, and 55.2%, 54.6%, and 56.8% on MATH500, respectively. By integrating our proposed RiemannInfer framework with these models, we observed consistent improvements across both benchmarks. Specifically, RiemannInfer enhanced the performance of Llama-3-405B to 93.5% on GSM8K and 58.4% on MATH500, corresponding to absolute increases of 1.9% and 3.2%. When applied to GPT-4o, RiemannInfer achieved accuracies of 95.8% and 57.9% on GSM8K and MATH500, representing gains of 2.6% and 3.3%, respectively. Similarly, DeepSeek-V2-400B, in combination with RiemannInfer, attained the highest accuracies of 94.8% on GSM8K and 58.9% on MATH500, with respective improvements of 2.3% and 2.1%. These results demonstrate that the RiemannInfer framework consistently boosts model performance on both mathematical reasoning and problem-solving tasks.

To validate the computational efficiency of RiemannInfer, we supplement the experiments with a runtime comparison against baseline models. Specifically, we report the average inference time per sample for each method on the GSM8K and MATH500 datasets. As shown in Table 2, RiemannInfer achieves competitive runtime performance compared to baseline methods. Overall, the average inference time of RiemannInfer is similar to or even slightly better than that of each baseline model. These results demonstrate that RiemannInfer maintains high computational efficiency while delivering improved reasoning accuracy.

**Table 2:** Runtime for baseline models and RiemannInfer.

Model	GSM8K(ms)	MATH500(ms)
Llama-3-405B	600	1100
GPT-4o	650	1000
DeepSeek-V2-400B	750	1200
Ours		
**RiemannInfer, Llama-3-405B**	550 $\downarrow$ **50**	1060 $\downarrow$ **40**
**RiemannInfer, GPT-4o**	550 $\downarrow$ **100**	950 $\downarrow$ **50**
**RiemannInfer, DeepSeek-V2-400B**	700 $\downarrow$ **50**	1100 $\downarrow$ **100**

### Out of domain results

The results of out-of-domain evaluation are shown in Table 3 and 4. Overall, the RiemannInfer trained on both the GSM8K and MATH500 datasets achieves competitive out-of-domain accuracy through iterative refinement, demonstrating refinement capabilities that extend beyond the original mathematical training domains. For the StrategyQA and AGIEval benchmarks, the RiemannInfer model trained on MATH500 outperforms its counterpart trained on GSM8K, exhibiting significant improvements with iterative refinement. Specifically, accuracies increase from 62.2% to 63.8% on StrategyQA and from 81.5% to 84.6% on AGIEval. These results highlight the robustness of the RiemannInfer approach.

**Table 3:** Model accuracy of StrategyQA.

Model	Acc.
Llama-3-405B	58.9%
GPT-4o	67.5%
DeepSeek-V2-400B	63.2%
Ours	
**RiemannInfer, GSM8K**	60.2%
-Iterative Refine	62.7% $\uparrow$ **2.5%**
**RiemannInfer, MATH500**	62.2%
-Iterative Refine	63.8% $\uparrow$ **1.6%**

**Table 4:** Model accuracy of AGIEval.

Model	Acc.
Llama-3-405B	78.3%
GPT-4o	84.5%
DeepSeek-V2-400B	77.5%
Ours	
**RiemannInfer, GSM8K**	78.7%
-Iterative Refine	80.1% $\uparrow$ **1.4%**
**RiemannInfer, MATH500**	81.5%
-Iterative Refine	84.6% $\uparrow$ **3.1%**

### Albation study

To further demonstrate the effectiveness of RiemannInfer, specifically, the feasibility and efficacy of constructing a Riemannian manifold from the hidden state of the Transformer, we conduct an ablation study by designing a linear model (LinearInfer) based on the hidden states as a control group. The experimental results are presented in Table 5. As shown, models constructed using the linear approach not only exhibit a substantial decrease in reasoning accuracy but also display an unstable performance trend. The accuracy of LinearInfer applied to various models consistently falls below 70%, resulting in a significant performance gap compared to RiemannInfer. These findings indicate that the manifold structure of RiemannInfer plays an essential and effective role in reasoning tasks.

**Table 5:** Model accuracy of GSM8K and MATH500.

Model	GSM8K	MATH500
Llama-3-405B	91.6%	55.2%
GPT-4o	93.2%	54.6%
DeepSeek-V2-400B	92.5%	56.8%
**LinearInfer, Llama-3-405B**	67.6% $\downarrow$ **24.0%**	28.6% $\downarrow$ **26.6%**
**LinearInfer, GPT-4o**	69.5% $\downarrow$ **23.7%**	30.5% $\downarrow$ **24.1%**
**LinearInfer, DeepSeek-V2-400B**	68.8% $\downarrow$ **23.7%**	30.6% $\downarrow$ **26.2%**
Ours		
**RiemannInfer, Llama-3-405B**	93.5% $\uparrow$ **1.9%**	58.4% $\uparrow$ **3.2%**
**RiemannInfer, GPT-4o**	95.8% $\uparrow$ **2.6%**	57.9% $\uparrow$ **3.3%**
**RiemannInfer, DeepSeek-V2-400B**	94.8% $\uparrow$ **2.3%**	58.9% $\uparrow$ **2.1%**

## Conclusion

In this paper, we propose RiemannInfer, a novel framework that leverages Riemannian manifolds to compute the inference work of the Transformer and achieve path planning for the inference process. By analyzing the spatial representation theory of Transformer hidden states and the geometric interpretation of the attention mechanism, we map the inference process onto a defined Riemannian manifold and provide methods for calculating the metric tensor, geodesics, and curvature on the manifold. Based on the fact that inference leads to changes in relative information entropy, and by examining the physical implications of geodesics and curvature on the manifold, we propose a method for calculating inference work on the manifold. Consequently, the path of minimal inference work can be determined, endowing the inference process of the Transformer with physical significance and enabling quantification, thus addressing the limitations of evaluating inference processes solely through probability distributions. Extensive experiments comparing RiemannInfer with other inference methods across multiple datasets demonstrate that RiemannInfer outperforms competing models in both computational efficiency and accuracy, underscoring its practicality and superiority.

RiemannInfer represents a significant step forward in advancing LLMs towards manifold-based research, marking a convergence between AI and the physical sciences. While RiemannInfer provides preliminary insights into the physical significance of the Transformer, the computation of geodesics, curvature, and inference work remains approximate and lacks the comprehensive formalism akin to established physical theories. Furthermore, reflecting on the attention mechanism of the Transformer, alternative structures may exist that better align with its intrinsic physical interpretation. In summary, our future work will focus on addressing these two aspects, aiming to contribute to the integration of artificial intelligence technologies with the domain of physics.

## Data Availability

The data that support the findings of this study are available from the corresponding author upon reasonable request.

## References

[1] He, J., Xiao, S., Huang, S., Li, J. & Wang, D. Mokan: A multi-omics data analysis framework using kolmogorov-arnold networks. *IEEE J. Biomed. Health Inform*, pp 1-11 (2025). 10.1109/JBHI.2025.3596817 40788803

[2] Achiam, J. et al. Gpt-4 technical report. arXiv preprint arXiv:2303.08774 (2023).

[3] Anil, R. et al. Palm 2 technical report. arXiv preprint arXiv:2305.10403 (2023).

[4] Wang, Q., Wang, Z., Su, Y., Tong, H. & Song, Y. Rethinking the bounds of llm reasoning: Are multi-agent discussions the key? arXiv preprint arXiv:2402.18272 (2024).

[5] Feng, G. et al. Towards revealing the mystery behind chain of thought: a theoretical perspective. *Adv. Neural Inf. Process. Syst.* **36**, 70757–70798 (2023).

[6] Wei, J. et al. Chain-of-thought prompting elicits reasoning in large language models. *Adv. Neural Inf. Process. Syst.* **35**, 24824–24837 (2022).

[7] Yao, S. et al. Tree of thoughts: Deliberate problem solving with large language models. *Adv. Neural Inf. Process. Syst.* **36**, 11809–11822 (2023).

[8] Besta, M. et al. Graph of thoughts: Solving elaborate problems with large language models. *Proc. AAAI Conf. Artif. Intell.* **38**, 17682–17690 (2024).

[9] Mishra, V. et al. Investigating the shortcomings of llms in step-by-step legal reasoning. arXiv preprint arXiv:2502.05675 (2025).

[10] Blair-Stanek, A., Holzenberger, N. & Van Durme, B. Can gpt-3 perform statutory reasoning? In: *Proceedings of the Nineteenth International Conference on Artificial Intelligence and Law*, pp. 22–31 (2023).

[11] Qiao, S. et al. Reasoning with language model prompting: A survey. arXiv preprint arXiv:2212.09597 (2022).

[12] Benfenati, A. & Marta, A. A singular riemannian geometry approach to deep neural networks i. theoretical foundations. *Neural Netw.* **158**, 331–343. 10.1016/j.neunet.2022.11.022 (2023). 36509003 10.1016/j.neunet.2022.11.022

[13] Gruffaz, S. & Sassen, J. Riemannian metric learning: Closer to you than you imagine. arXiv preprint arXiv:2503.05321 (2025).

[14] McDuff, D. et al. Towards accurate differential diagnosis with large language models. *Nature* **642**, 451–457 (2025). 40205049 10.1038/s41586-025-08869-4 PMC12158753

[15] Lu, J. et al. Where views meet curves: Virtual anchors for hyperbolic multi-view graph diffusion. In: *Proceedings of the 33rd ACM International Conference on Multimedia*, pp. 2131–2140 (2025).

[16] Vaswani, A. et al. Attention is all you need. *Adv. Neural Inf. Process. Syst.* **30**, (2017).

[17] Beltagy, I., Peters, M.E. & Cohan, A. Longformer: The long-document transformer. arXiv preprint arXiv:2004.05150 (2020).

[18] Gao, L., Madaan, A., Zhou, S., Alon, U., Liu, P., Yang, Y., Callan, J. & Neubig, G. Pal: Program-aided language models. In: *International Conference on Machine Learning*, pp. 10764–10799 (PMLR, 2023).

[19] Guu, K., Lee, K., Tung, Z., Pasupat, P. & Chang, M. Retrieval augmented language model pre-training. In: *International Conference on Machine Learning*, pp. 3929–3938 (PMLR, 2020).

[20] Amizadeh, S., Palangi, H., Polozov, A., Huang, Y. & Koishida, K. Neuro-symbolic visual reasoning: Disentangling. In: *International Conference on Machine Learning*, pp. 279–290 (PMLR, 2020).

[21] Ganea, O., Bécigneul, G. & Hofmann, T. Hyperbolic neural networks. *Adv. Neural Inf. Process. Syst.* **31**, (2018).

[22] Nickel, M. & Kiela, D. Poincaré embeddings for learning hierarchical representations. *Adv. Neural Inf. Process. Syst.* **30**, (2017).

[23] Chami, I., Abu-El-Haija, S., Perozzi, B., Ré, C. & Murphy, K. Machine learning on graphs: A model and comprehensive taxonomy. *J. Mach. Learn. Res.* **23**(89), 1–64 (2022).

[24] Pan, Y.-T., Chou, J.-L. & Wei, C.-S. Matt: A manifold attention network for eeg decoding. *Adv. Neural Inf. Process. Syst.* **35**, 31116–31129 (2022).

[25] McInnes, L., Healy, J. & Melville, J. Umap: Uniform manifold approximation and projection for dimension reduction. arXiv preprint arXiv:1802.03426 (2018).

[26] Bécigneul, G. & Ganea, O.-E. Riemannian adaptive optimization methods. arXiv preprint arXiv:1810.00760 (2018).

[27] Wang, H. et al. Manifold-based verbalizer space re-embedding for tuning-free prompt-based classification. *Proc. AAAI Conf. Artif. Intell.* **38**, 19126–19134 (2024).

[28] Sun, L. et al. Motif-aware riemannian graph neural network with generative-contrastive learning. *Proc. AAAI Conf. Artif. Intell.* **38**, 9044–9052 (2024).

[29] Pope, P., Zhu, C., Abdelkader, A., Goldblum, M. & Goldstein, T. The Intrinsic Dimension of Images and Its Impact on Learning (2021). arXiv:2104.08894.

[30] Dijkstra, E.W. A note on two problems in connexion with graphs. In: *Edsger Wybe Dijkstra: His Life, Work, and Legacy*, pp. 287–290 (2022).

[31] Grattafiori, A., Dubey, A., Jauhri, A., Pandey, A. & Kadian, A. The Llama 3 Herd of Models arXiv:2407.21783 (2024).

[32] OpenAI, Achiam, J., Adler, S., Agarwal, S. & Ahmad, L. GPT-4 Technical Report arXiv:2303.08774 (2024).

[33] DeepSeek-AI, Guo, D., Yang, D., Zhang, H. & Song, J. DeepSeek-R1: Incentivizing Reasoning Capability in LLMs via Reinforcement Learning arXiv:2501.12948 (2025).

